# Microbial Variability in Zoo‐Housed Alaotran Gentle Lemurs: Host‐Associated Factors and Environmental Influences

**DOI:** 10.1002/ajp.70202

**Published:** 2026-08-26

**Authors:** Sara Fontani, Agnese Gori, Benedetta Cerasuolo, Aldo D'Alessandro, Sonia Renzi, Gale Glendewar, Rachel Cowen, Duccio Cavalieri, Stefano Vaglio

**Affiliations:** ^1^ Animal Behaviour & Wildlife Conservation Group, School of Pharmacy & Life Sciences University of Wolverhampton Wolverhampton UK; ^2^ Department of Biology University of Florence Florence Italy; ^3^ Department of Medical Parasitology and Infection Biology Swiss Tropical and Public Health Institute Allschwil Switzerland; ^4^ Durrell Wildlife Conservation Trust ‐ Jersey Zoo, Trinity, Jersey, Channel Islands; ^5^ University College – The Castle Durham University Durham UK

**Keywords:** fecal endocrinology, gut microbiota, *Hapalemur alaotrensis*, primate welfare and management, zoo biology

## Abstract

Primates worldwide face escalating ecological pressures, driving many species to rapid declines. Modern zoos play an essential role by maintaining viable populations and improving welfare through research‐driven management. However, gut–microbiome interactions in captive primates remain poorly understood. Our study investigated how host‐specific traits influence fecal microbiota composition, whether shared environments facilitate shared microbial signatures, and whether fluctuations in fecal cortisol and testosterone correspond to microbial changes. We also evaluated the combined use of hormonal and microbiota analyses as a noninvasive tool to support welfare and health monitoring in zoo‐housed *Hapalemur alaotrensis*. We monitored 4 groups (*n* = 8) housed across 4 European facilities, yielding 180 fecal samples across 3 collection phases. We characterized microbial communities through 16S rRNA gene sequencing following DNA extraction and quality checks, and quantified hormone metabolites using ELISA kits. We performed statistical analyses to evaluate the effects of facility, collection phase, and host‐specific traits on microbiota composition and hormone concentrations. Microbiota profiling revealed diverse bacterial communities, with facility‐associated differences representing the strongest and most consistent pattern, while effects of sex and scent enrichment were weak and inconsistent. Hormone concentrations did not vary across collection phases, although cortisol differed among facilities. No significant associations between hormone concentrations and microbiota profiles were detected. Given the small sample size, these findings should be considered exploratory, providing initial evidence of facility‐associated microbiome variation in zoo‐housed *H. alaotrensis*. Larger studies are needed to investigate its potential drivers and evaluate whether combined microbiome and endocrine assessments may contribute to primate welfare monitoring.

## Research Highlights

- Inter‐zoo differences emerged consistently across multiple microbiota analyses, suggesting an important role of environmental and husbandry‐related factors in shaping the gut microbiota of human‐managed *Hapalemur alaotrensis*.
- Sex and scent enrichment showed weak associations with gut microbiota, whereas age could not be evaluated because of the limited sample size.
- Integrating microbiome profiling with endocrine assessments represents a promising noninvasive approach for informing welfare monitoring and conservation management in endangered lemurs.

## Graphical abstract

Environmental variation is associated with gut microbiota differences in zoo‐housed endangered lemurs.

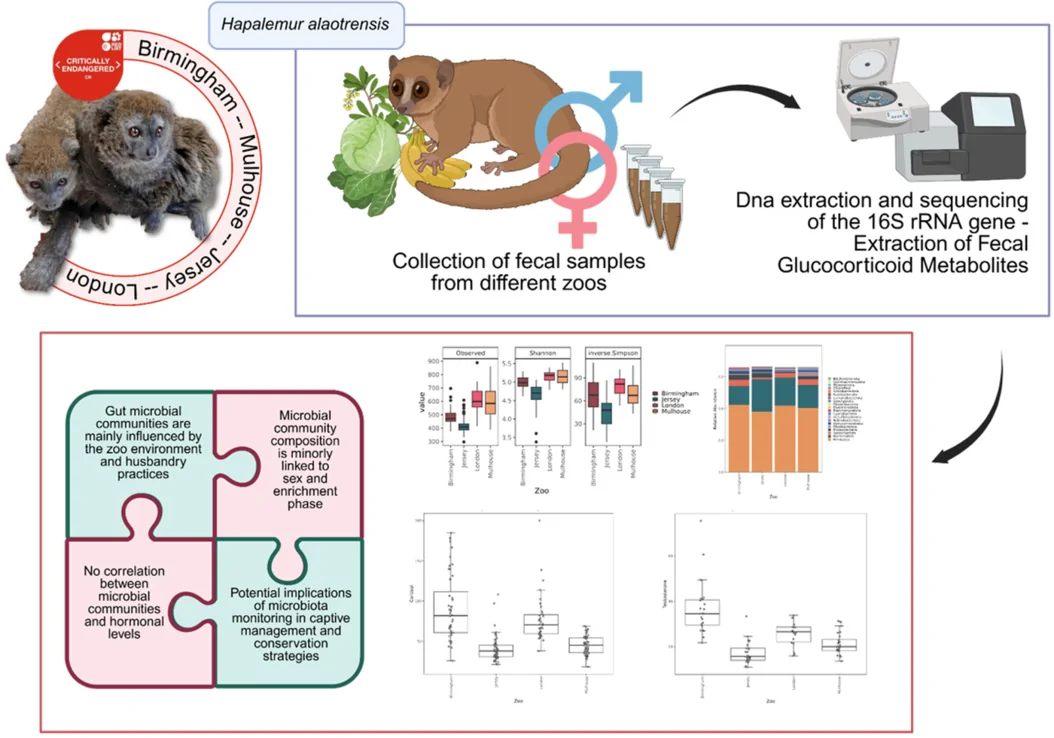

## 1 Introduction

Primates worldwide are currently subject to significant ecological pressures (Benchimol and Peres 2014; Estrada and Garber 2022; Estrada et al. 2017; Fernández et al. 2022). Numerous species are critically endangered, with their populations experiencing rapid declines (Cardinale et al. 2012; Walsh et al. 2003). Species with small population sizes are particularly vulnerable to demographic stochasticity and local disasters, which increases their risk of extinction compared to species with larger populations (Purvis et al. 2000). The conservation of primates has thus become a complex, global challenge that necessitates diverse strategies to effectively monitor and safeguard primate populations (Estrada and Garber 2022). Contemporary wildlife conservation efforts incorporate both in situ strategies and ex situ measures, like captive breeding and reintroduction programs (Ferrie 2017; Kaltenborn and Linnell 2021; Stumpf et al. 2016), where zoos and other breeding facilities outside natural habitats serve as genetic reserves, aiming to maintain genetic diversity within these populations (Ferrie 2017; Russello and Amato 2004). In this context, research focused on captive animal welfare is essential to enhance husbandry and management standards, improve breeding programs and maximize the success of reintroduction efforts (Fernandez and Martin 2021; Greggor et al. 2018; Kemp et al. 2017).

In the last few years, the analysis of primate microbiomes has emerged as a promising set of tools with significant potential to monitor and enhance captive animal well‐being and, by extension, conservation efforts (Diaz and Reese 2021; Quiroga‐González et al. 2021; Stumpf et al. 2016).

Primate bodies harbor vast numbers of microbial cells, amounting to trillions (Bianconi et al. 2013; Peterson et al. 2009; Qin et al. 2010). These microbial communities exhibit extensive genetic diversity, with their composition varying across species, individuals, specific body sites, age, and life stages (Aagaard et al. 2012; Asangba et al. 2022; Costello et al. 2009; K. Li et al. 2012; Pereira et al. 2024; Sun et al. 2018). Additionally, these communities are influenced by host dietary habits, habitat characteristics, and environmental changes (Barelli et al. 2015; Ma et al. 2023; Nova et al. 2022). The microbiome is crucial for digestion and nutrition, profoundly impacting the host's ability to process and absorb nutrients (Afzaal et al. 2022; Rowland et al. 2018; Samuel et al. 2008). Microbes residing in the large intestine play a crucial role in making nutrients more accessible by breaking down complex fibers and starches. This process aids in nutrient absorption and leads to the production of short‐chain fatty acids (SCFAs), which are an important energy source for the host (Oliphant and Allen‐Vercoe 2019; Samuel et al. 2008; Valdes et al. 2018). Therefore, microbiome studies also help pinpoint the causes of diet‐related illnesses often seen in captivity (Martínez‐Mota et al. 2020). For example, florivorous primates, such as Asian colobines (e.g., *Pygathrix nemaeus*) and woolly spider monkeys (*Brachyteles arachnoides*), are particularly challenging to sustain in captivity (Janssen 1994; Milton 1986), as these species frequently experience high mortality rates, with diarrhea being a common cause of death (Davies and Oates 1994; Pope 1998).

The specialized digestive systems of many florivorous primates include either a large, multichambered stomach housing anaerobic cellulolytic bacteria that break down secondary toxins and high‐cellulose foods or an enlarged cecum or colon where microbial fermentation occurs (Davies and Oates 1994; Liu et al. 2022). Considering the impact of diet on gut microbiome composition, the coadaptation between host and microbiome, and the crucial role the gut microbiome plays in digesting fibrous and toxin‐laden diets, it is not surprising that, in many studies, captivity is associated with reduced microbiome diversity and subsequent health issues (Amato et al. 2013; McKenzie et al. 2017; Xia et al. 2022).

Besides their role in nutrition, microbial communities are vital for immune defense, contributing to the development and regulation of the host's immune system by influencing both innate and adaptive immunity (Costello et al. 2009; Dominguez‐Bello et al. 2010; Graham and Xavier 2023; Kogut et al. 2020; Schachtschneider et al. 2013).

In the past few years, growing attention has shifted to how gut microbes also influence neuroendocrine pathways, highlighting their involvement in bidirectional communication between the gastrointestinal tract and the central nervous system. There is accumulating evidence that neuroendocrinological dysregulation may also be involved in the potential pathway linking psychosocial stressors, the enteric microbiome, proinflammatory cytokines, immunity and social behavior (Liu 2017; Martin et al. 2011; So and Savidge 2022), with microbiota playing a significant role in the gut–brain axis signaling, extending the commonly used terminology to the “microbiota–gut–brain axis” (So and Savidge 2022). Disruptions in the gut microbiota can affect the stress response and behavior, while steroids can, in turn, impact the composition of the gut microbiota (Tetel et al. 2018). Previous studies also demonstrated that stress‐induced cortisol production is associated with disruption of the gut microbiome, which may, in turn, contribute to the adverse effects of stress on human and animal health (Uren Webster et al. 2020; Vodička et al. 2018; Wiley et al. 2017). Martínez‐Mota et al. (2020) found that the activation of the hypothalamic–pituitary–adrenal (HPA) axis, a physiological response sensitive to environmental stressors like habitat disturbance, plays a role in shaping the gut microbiome of black howler monkeys (*Alouatta pigra*) living in disturbed forests. Additionally, as highlighted in a recent review by D'Afflitto et al. (2022), there is a link between sex hormones and the composition and diversity of the gut microbiota in humans, which may help explain the sex‐based differences observed in disease development. Nevertheless, limited information is available on the influence of hormones on the enteric nervous system and gastrointestinal function (So and Savidge 2022), especially regarding nonhuman primates (Clayton et al. 2018).

### 1.1 Study Species

This study focuses on the Alaotran gentle lemur (*Hapalemur alaotrensis*), a small‐bodied lemur species native to the wetlands around Madagascar's Lake Alaotra. It is the only lemur species restricted exclusively to wetland ecosystems (Mutschler 2002). Severe habitat degradation, hunting pressure, and an extremely limited distribution have led to a dramatic decline from approximately 7500 individuals (1994) to around 2500 (2018) (Waeber et al. 2018). Listed among the World's 25 Most Endangered Primates (2018–2020) (Reibelt et al. 2019), the species is difficult to maintain and breed under human care.

Studies of their natural diet have shown that Alaotran gentle lemurs are highly selective feeders, relying almost entirely on four plant species from the Cyperaceae and Poaceae families (Mutschler 1999). This reliance on a restricted diet is unique among primates, with no other known species subsisting almost exclusively on such a narrow range of food sources (Tan 2006). For instance, captive Alaotran gentle lemurs have shown various gastrointestinal, renal, and integumentary pathologies, with diet suspected as a primary or contributing factor (Barbon et al. 2016).

This species was chosen for our study, in collaboration with the European Association of Zoos and Aquaria (EAZA) Ex‐situ Programme (EEP) Coordinator, who identified *H. alaotrensis* as a priority species for evidence‐based management improvements.

### 1.2 Aims of the Study

This study builds upon a previously published data set on the same study populations, in which behavioral and endocrine responses to a new scent enrichment protocol on the well‐being and reproductive potential of captive Alaotran gentle lemurs were investigated (Fontani et al. 2025). While the samples and individuals overlap, the present manuscript addresses a distinct research question by focusing on gut microbiota composition and its ecological and physiological correlates. In particular, the aims of this study were:

a. to investigate how fecal microbiome composition differs according to host‐specific characteristics and whether a shared environment facilitates microbial similarity among co‐housed individuals;
b. to examine associations between hormonal changes (cortisol in both sexes and testosterone in males) with changes in gut microbiota diversity and abundance, to better understand bidirectional signaling between the gut and the central nervous system; and
c. to explore the potential of the simultaneous analysis of hormone levels and gut microbiota as a noninvasive tool for primate welfare assessment, and to evaluate the potential use of the microbiota to improve animal health.

## 2 Methods

### 2.1 Study Subjects and Housing

The data set analyzed here derives from the same individuals and fecal samples collected during the study described in Fontani et al. (2025), which focused on behavioral and endocrine responses to scent enrichment.

We studied four captive pairs of Alaotran gentle lemurs (4 males and 4 females; *n* = 8) housed at Birmingham Wildlife Conservation Park (UK), Parc Zoologique & Botanique de Mulhouse (France), Jersey Zoo (Channel Islands), and ZSL London Zoo (UK). All subjects in this research are part of a controlled EEP aimed at maintaining healthy, thriving populations and preserving genetic diversity within European zoological institutions. These individuals were specifically selected to assess the potential of olfactory enrichment in stimulating reproductive‐related behaviors in pairs biologically capable of reproducing but showing limited or absent mating activity. They were also housed under suitable conditions for the study (i.e., good visibility of the animals and their latrines).

All individuals followed a regulated diet plan (Table S1), were under continuous veterinary supervision, and remained in good health throughout the study. All pairs were kept in continuous full contact in natural outdoor enclosures, with access to indoor areas maintained at 25°C–28°C during the colder months. No other species were housed within these enclosures, and no additional enrichment was provided during the data collection period.

### 2.2 Scent Enrichment

As detailed in Fontani et al. (2025), we opted to test a new scent enrichment based on the female's fertile signal, previously collected, analyzed, and resynthesized in the lab. The scent enrichment consisted of a mixture of four chemical compounds (2‐heptanone, 3‐heptanone, 3‐octanone, and 4‐methyl‐3‐hexanone) we had previously identified as markers of the fertile window in female Alaotran gentle lemurs (Fontani et al. 2022).

**Figure 1:**
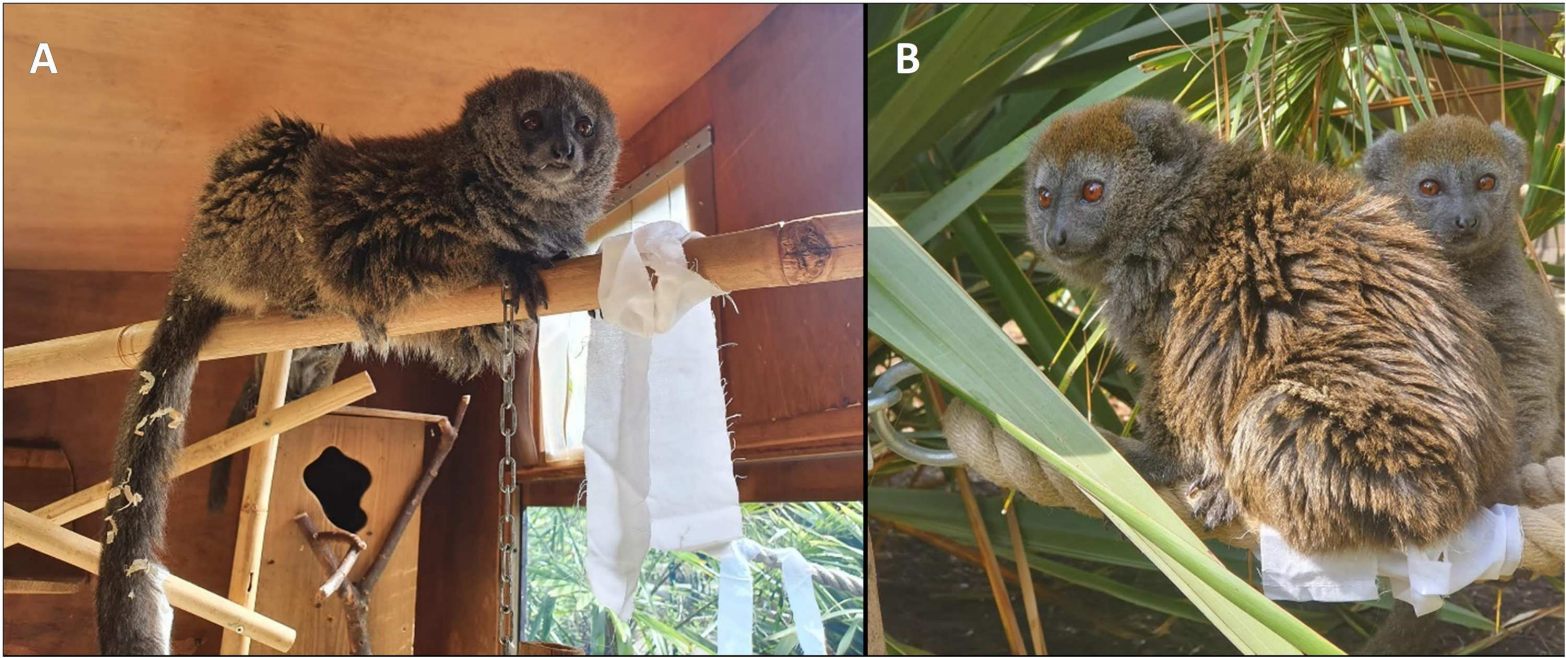
Male gentle lemur interacting with scent enrichment in the indoor enclosure (A), and both individuals in the outdoor enclosure (B) at Birmingham Wildlife Conservation Park (UK). Photos by Georgia Callagan.

We presented the enrichment to the study subjects following the protocol established in our previous research (Vaglio et al. 2021). Briefly, we used white cotton strips (75 × 5 cm) as enrichment items, placing six scented and two unscented strips on the indoor and outdoor climbing frames (Figure 1A,B). We soaked each scented strip with 20 drops of the mixture, previously diluted with water, and prepared fresh ones every day. At the end of each observation period, we removed all strips, and we randomized their placement daily to minimize habituation (Costantini et al. 2024).

### 2.3 Study Protocol

Fecal samples were collected from June 2022 to February 2023, as part of the study described in Fontani et al. (2025). The study was structured into three phases: pre‐enrichment (10 days before the enrichment), enrichment (6 days of enrichment), and post‐enrichment (10 days after the enrichment). To minimize habituation to the scent stimuli, the enrichment phase was divided into two 3‐day periods separated by a 1‐day interval (Elwell et al. 2024; Wowk and Behie 2023). Samples collected during the interval day were included within the enrichment phase. Fecal samples were collected daily across 6 days each week, ensuring collection only when defecation was observed and the specific individual was identified. We then analyzed these samples for cortisol levels in both males and females, as well as testosterone levels in males, with the expectation that the female‐derived scent enrichment would elevate male testosterone levels. Additionally, we examined fecal microbiome diversity and abundance at the amplicon sequence variant (ASV) level.

### 2.4 Fecal Samples Collection and Measurements

We collected a total of 180 fecal samples throughout the study period (Table 1). We gathered the samples every morning, immediately following defecation. To account for diurnal secretion patterns of hormones like cortisol and testosterone, which can be detected in fecal samples (particularly in small‐bodied species), we restricted the sampling to approximately the same time each day (Hodges and Heistermann 2011). After collection, we separated a small portion (approximately 0.5 g) of the fecal matter and placed it into 1.5 mL Eppendorf Safe‐Lock micro test tubes (Scientific Laboratory Supplies, UK), each containing 250 µL of RNAlater solution (Scientific Laboratory Supplies, UK), to stabilize and protect cellular DNA. The remaining portion of the fecal sample was placed in 50 mL Eppendorf Conical Tubes (Scientific Laboratory Supplies, UK). All samples were immediately stored on‐site in a −20°C freezer. At the end of the study period, we transferred the RNAlater‐treated samples to the Microbiology Laboratory at the University of Florence for molecular diagnostics, and the remaining samples to the Rosalind Franklin Science Centre at the University of Wolverhampton for endocrinological analyses. The samples were transported in insulated coolers with ice packs to prevent any risk of thawing.

**Table 1:** Details of the samples collection.

Period of sampling	Zoo	Individual	Age at study start (years)	Experimental phase	Fecal samples (number)
27/06/22–10/08/22	Birmingham	Bozy (F)	12	Pre	8
				Enr	7
				Post	8
		Zoma (M)	15	Pre	9
				Enr	6
				Post	6
25/07/22–26/08/22	Mulhouse	Manon (F)	12	Pre	10
				Enr	7
				Post	10
		Kwic (M)	8	Pre	11
				Enr	7
				Post	10
19/09/22–21/10/22	Jersey	Bala (F)	17	Pre	7
				Enr	7
				Post	7
		Brian (M)	13	Pre	10
				Enr	7
				Post	9
09/01/23–10/02/23	London	Hazo (F)	3	Pre	7
				Enr	4
				Post	5
		Rocky (M)	15	Pre	8
				Enr	4
				Post	6

Abbreviations: Enr, enrichment phase; F, female; M, male; Post, post‐enrichment phase; Pre, pre‐enrichment phase.

### 2.5 Microbiota Analysis

#### 2.5.1 DNA Extraction and Sequencing

Once defrosted, we extracted DNA from all samples using the DNeasy PowerSoil Kit (Qiagen, Hilden, Germany) according to the manufacturer's protocol. We assessed extraction success by electrophoresis on a 1% agarose gel. We measured DNA concentration using the Qubit 4 Fluorometer (Thermo Fisher Scientific, Waltham, MA, USA) with the Qubit dsDNA High Sensitivity assay, and we used these measurements to prepare the samples for subsequent analyses. We amplified the V3–V4 hypervariable regions of the 16S rRNA gene (341 f: 5′‐CCTACGGGNGGCWGCAG‐3′ and 805r: 5′‐GACTACNVGGGTWTCTAATCC‐3′) on each sample for bacterial community profiling, using specific primer sets supplied with Illumina overhang adapters (Takahashi et al. 2014; White et al. 1990). The amplicon library was prepared following Illumina protocols (Illumina 2013), and we performed paired‐end 2 × 300 bp sequencing on an Illumina MiSeq instrument (Illumina Inc., San Diego, CA, USA) using the MiSeq Reagent Kit v3 (600‐cycle) (Bacci et al. 2023; Nel Van Zyl et al. 2022).

#### 2.5.2 Post‐Run Analyses

We preprocessed and cleaned the demultiplexed sequences obtained from sequencing using Cutadapt v.4.10 and the Divisive Amplicon Denoising Algorithm (DADA2) v.1.16 (Callahan et al. 2016) R packages. We removed primers using Cutadapt under default settings. We filtered sequences with the filterAndTrim function of DADA2, using a maximum error rate of 2 for forward reads and 3 for reverse reads. We removed chimeras with the “removeBimeraDenovo” function from the same package. We then trimmed sequences at positions 280 (forward) and 220 (reverse), denoised them, merged them, and inferred ASVs. For taxonomic assignment, we mapped the sequences to the SILVA SSU r138 database using the DECIPHER v.3.0 package. We performed subsequent analyses using the phyloseq v.1.41.1 (McMurdie and Holmes 2013), microbiome v.1.26.0 (Lahti and Shetty 2017), vegan v.2.6 (Oksanen et al. 2024), and tidyverse v.2.0.0 (Wickham et al. 2019) packages. We manually calculated alpha diversity values and performed a *t*‐test to investigate possible differences. We explored beta‐diversity with a Redundancy Analysis (RDA) approach. We evaluated the influence of sample distribution across groups on microbial communities with a PERMANOVA analysis using the “adonis2” command from the vegan package, using the Subject Identity (ID) as a blocking factor to avoid pseudoreplication. To detect significant alterations in ASV abundance across groups, we conducted differential abundance analysis with DESeq. 2 v.1.44.0 (Love et al. 2014), applying a *q* value threshold of 0.05 with a Likelihood Ratio Test (LRT) approach, using sfType = “poscounts” and fitType = “local”.

### 2.6 Hormone Analyses

We used a freeze‐drying machine (Christ R, Beta 1–8 LSC plus, Osterode am Harz, Germany) to lyophilize the fecal samples for 72 h. After lyophilization, we ground the samples into a fine powder using a pestle and mortar. We then sieved the powdered fecal material through a stainless‐steel strainer with a 250‐micron aperture to separate the fecal residue from any fibrous material. For the extraction process, we followed the protocols established in our earlier studies (Elwell et al. 2024; Fontani et al. 2022). Specifically, we extracted 0.05–0.1 g of fecal powder in 3 mL of 80% methanol using 15 mL plastic tubes and vortexed the mixture for 15 min with a multitube vortexer (Grant Instruments R, Multi‐Vortexer V‐32, Cambridge, UK). After centrifuging the samples for 20 min at 3300*g*, we stored the supernatant at −20°C.

In our analysis of fecal hormones, we considered the timing of hormone metabolite excretion relative to the production and circulation of the native hormones (Hodges and Heistermann 2011; Wheeler et al. 2013). We measured fecal cortisol and testosterone concentrations using commercially available enzyme‐linked immunosorbent assay (ELISA) kits (Enzo Life Sciences Cortisol, ADI‐900‐071, New York, USA, and DetectX Testosterone K032‐H5W, Arbor Assays R, USA) following the manufacturers' instructions. Before analysis, we diluted all samples 1:1 with the assay buffer provided in the kits. We assayed all standards and fecal samples in duplicate, re‐analyzing any samples with a coefficient of variation (CV) exceeding 15% (Macagno et al. 2020). We randomly distributed the samples across the assay plates and analyzed the resulting data using a four‐parameter logistic fitting program (MyAssays R, Brighton, UK). We expressed hormone concentrations as pg/mg. The mean intra‐assay CV for cortisol, assessed using four quality control samples (two males and two females) with eight replicates on a single assay plate, was 7.77% ± 1.27%. For testosterone, evaluated on three control samples (all males), the mean intra‐assay CV was 9.35% ± 2.57%. The mean inter‐assay CV, tested on the same samples with four replicates across three assay plates, was 15.04% ± 5.21% for cortisol and 5.96% ± 1.42% for testosterone.

#### 2.6.1 Statistical Analyses of Hormone Data

We conducted statistical analyses in R, version 4.4.1 (R Core Team 2020). As described in Fontani et al. (2025), we investigated the impact of the enrichment phase (pre‐, during, and post‐enrichment) on fecal cortisol concentration by fitting a Linear Mixed Model (LMM) using the lmer() function from the lme4 package (Bates et al. 2015). To meet the assumption of normally distributed residuals, cortisol concentration was log‐transformed (base 10) and used as the dependent variable. The model included zoo, enrichment phase, subject sex, and the interaction between enrichment phase and sex as fixed effects. Subject Identity (ID) was included as a random effect. Initially, we specified both random intercepts and slopes for enrichment phase by subject ID; however, this led to a singular fit. To address this, we simplified the model by retaining only random intercepts for subject ID. We conducted a similar analysis to assess the effects of the enrichment phase on testosterone concentration.

For the present study, we extended the hormone analyses to evaluate the effects of zoo and sex on cortisol and testosterone concentrations. For cortisol, subject ID was used as a random effect to avoid the risk of pseudoreplication, while zoo, enrichment phase, and sex were used as fixed effects. Since testosterone was measured exclusively in male fecal samples, and each zoo included only one male individual, the model was run without random effects. This model was fitted as a linear regression model (LM) using the lm() function from the stats package (R Core Team 2020). To assess the robustness of the obtained results, the same model was run on a simplified data set, obtained by averaging replicate cortisol and testosterone values.

## 3 Results

### 3.1 Metagenomics Results

Sequencing produced a total of 15,828,689 reads. After sequence cleaning, merging, and chimera removal, the retained reads were 74.68% (11,939,780 reads). Of all the 180 samples analyzed, one was excluded from the subsequent analyses due to its low number of reads.

The 16S rRNA gene sequencing produced 12,223 ASVs. Most variants were assigned to the Bacteria domain (10,346 ASVs, 84.64% of the total ASVs detected), and a marginal presence of Archaea (19 ASVs, 0.16% of the total ASVs detected) and Eukaryotic domain (1 ASV, 0.01% of the total ASVs detected) was found. A total of 1857 ASVs were not assigned to any domain. After removing the ASVs not assigned to the Bacteria domain and the singletons, the final number of ASVs was 10,316.

**Figure 2:**
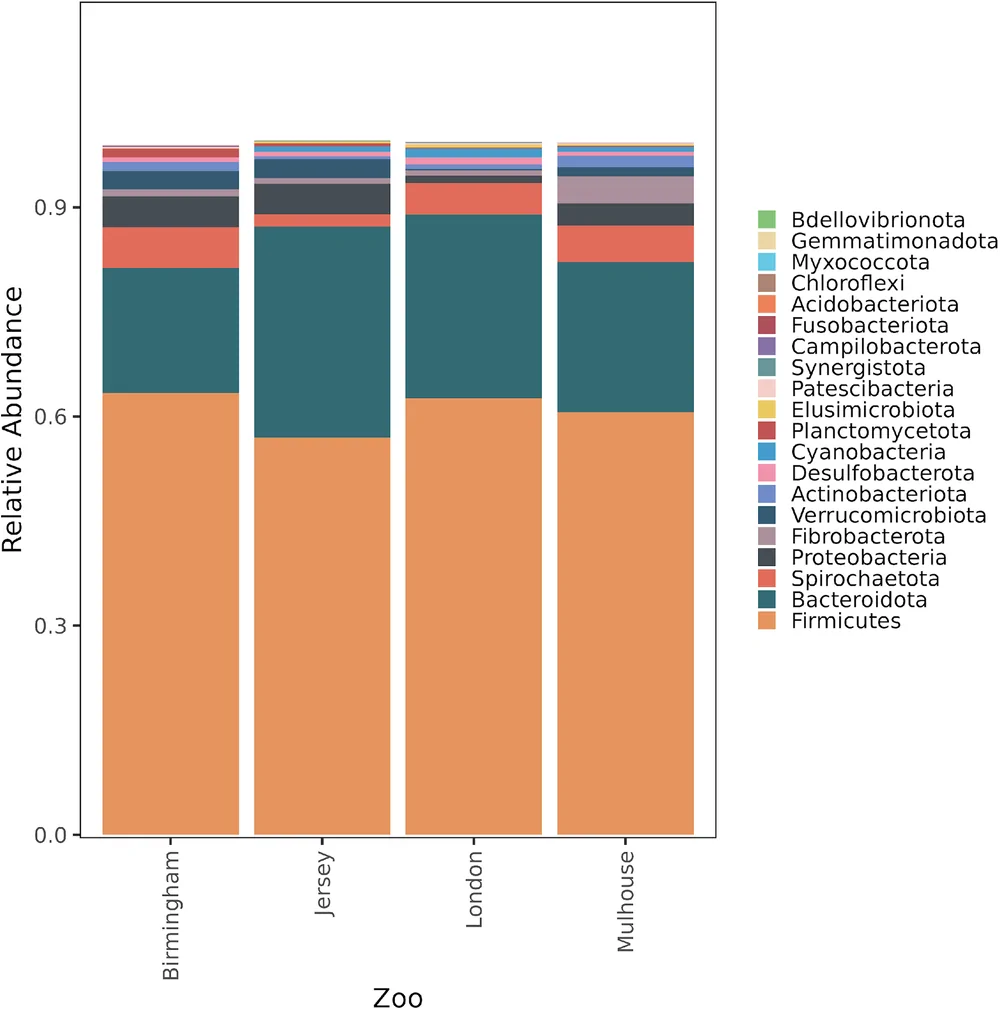
Mean relative abundance of bacterial phyla in the fecal microbiota of *Hapalemur alaotrensis* across the four study zoos.

At the phylum level, gut microbial communities were dominated by Firmicutes and Bacteroidota across all zoological institutions (Figure 2). Lower relative abundances were observed for Spirochaetota, Proteobacteria, and Fibrobacterota, whereas the remaining phyla represented only a minor component of the bacterial community.

**Figure 3:**
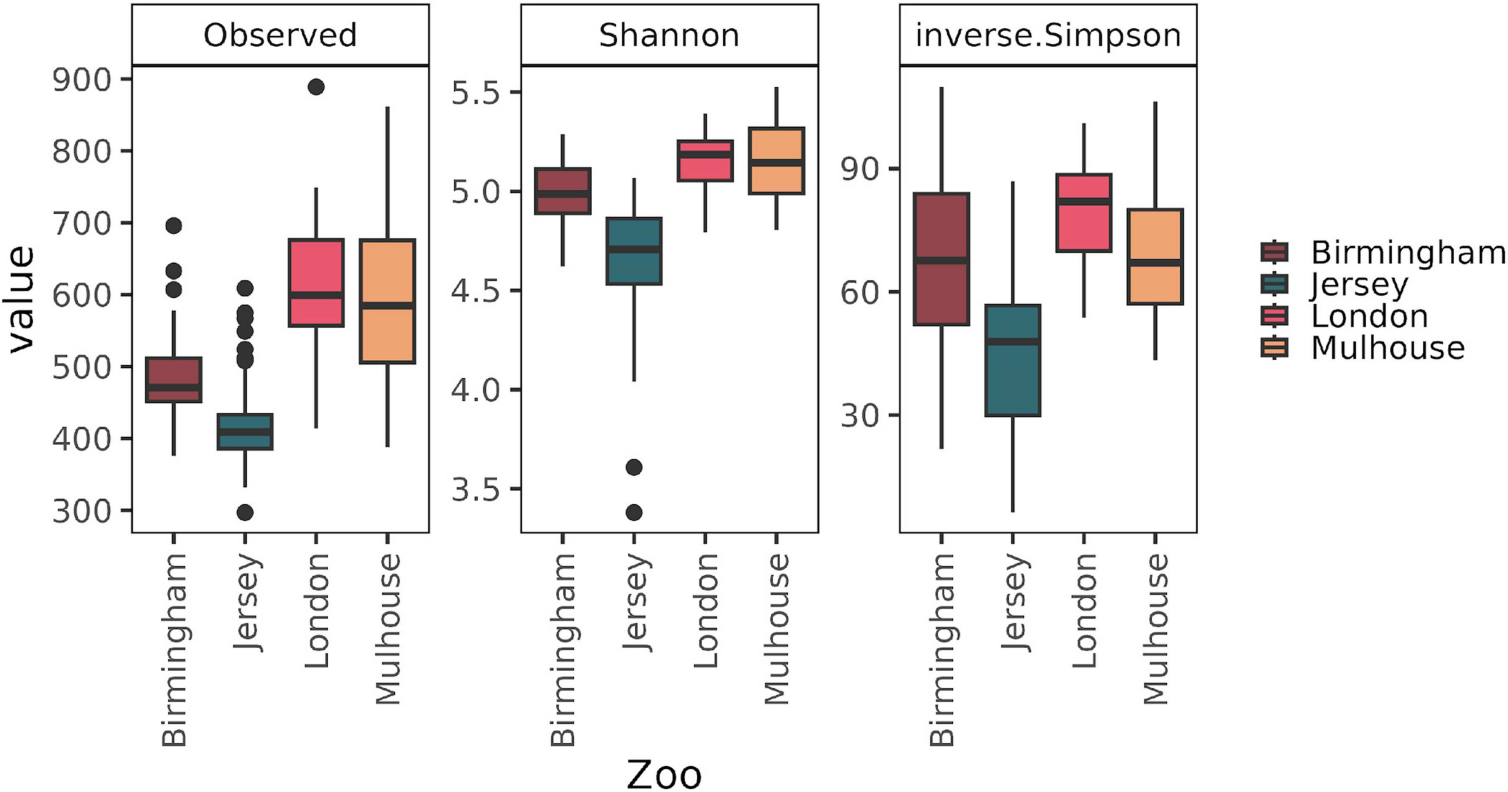
Alpha diversity indices (Observed richness, Shannon, and Inverse Simpson) of the fecal microbiota of *Hapalemur alaotrensis* across the four study zoos. Boxplots show the distribution of diversity values for individuals housed in Jersey, Birmingham, London, and Mulhouse.

Gut microbial richness and diversity varied across zoological institutions. Alpha diversity analyses (Observed richness, Shannon, and Inverse Simpson indices) revealed significant differences in gut microbiota diversity among lemurs from the four zoos, with London and Mulhouse individuals showing higher richness and diversity compared to Jersey and Birmingham (Figure 3).

Statistical analysis revealed that sex and the environment had a significant effect on Shannon index (*p* = 4.96*e^−06^ and *p* = 2.2*e^−16^, respectively) and environment had a significant influence on the Simpson index (*p* = 0.028), while Observed index was not affected by either of the two factors. Pairwise comparison between zoos showed that Jersey and London were the only significantly different ones, with a *p* value of 0.03.

#### 3.1.1 Community Composition and Differences

**Figure 4:**
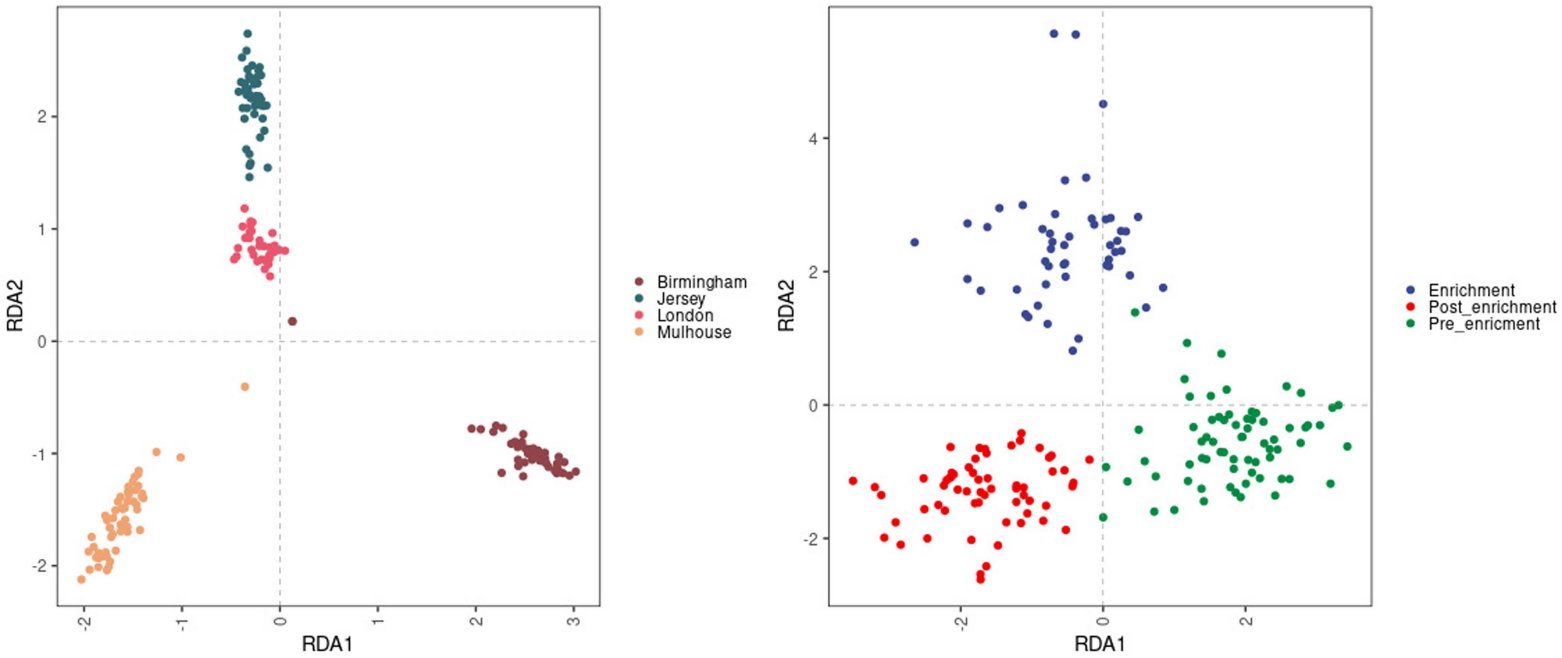
Redundancy Analysis (RDA) based on Bray–Curtis dissimilarities, illustrating the beta diversity of fecal microbiota in *Hapalemur alaotrensis*. The plots represent the results of RDA when using the zoos as fixed factor (left), and when using time points as fixed factor while zoos as random factor (right). Each point represents a single fecal sample, colored by zoo of origin (left) and time point (right). Samples cluster distinctly according to zoo, indicating strong site‐specific microbial signatures.

The beta diversity analysis (Figure 4), performed on the sequences, showed that the factor most strongly differentiating our sample groups was the environment, that is, the zoo. These differences were demonstrated to be significant by the results from the PERMANOVA analysis, which showed a high influence of the environment on microbial community composition (*R* ^2^ statistic = 0.304), with a pseudo‐*F* ratio of 26.478 and a *p* value of 0.001 (***), and a minor but significant influence of sex (*R* ^2^ = 0.02, *F* = 5.951, *p* = 0.001) and enrichment phase (*R* ^2^ = 0.02, *F* = 5.951, *p* = 0.001). However, when conditioning the model on the individual IDs, the only variable which explained additional variance is the enrichment phase. The gut bacterial composition of the samples from Birmingham Zoo is clearly separated from that of the other three zoos, and a greater proximity is also observed between the groups of samples from London and Jersey zoos.

**Figure 5:**
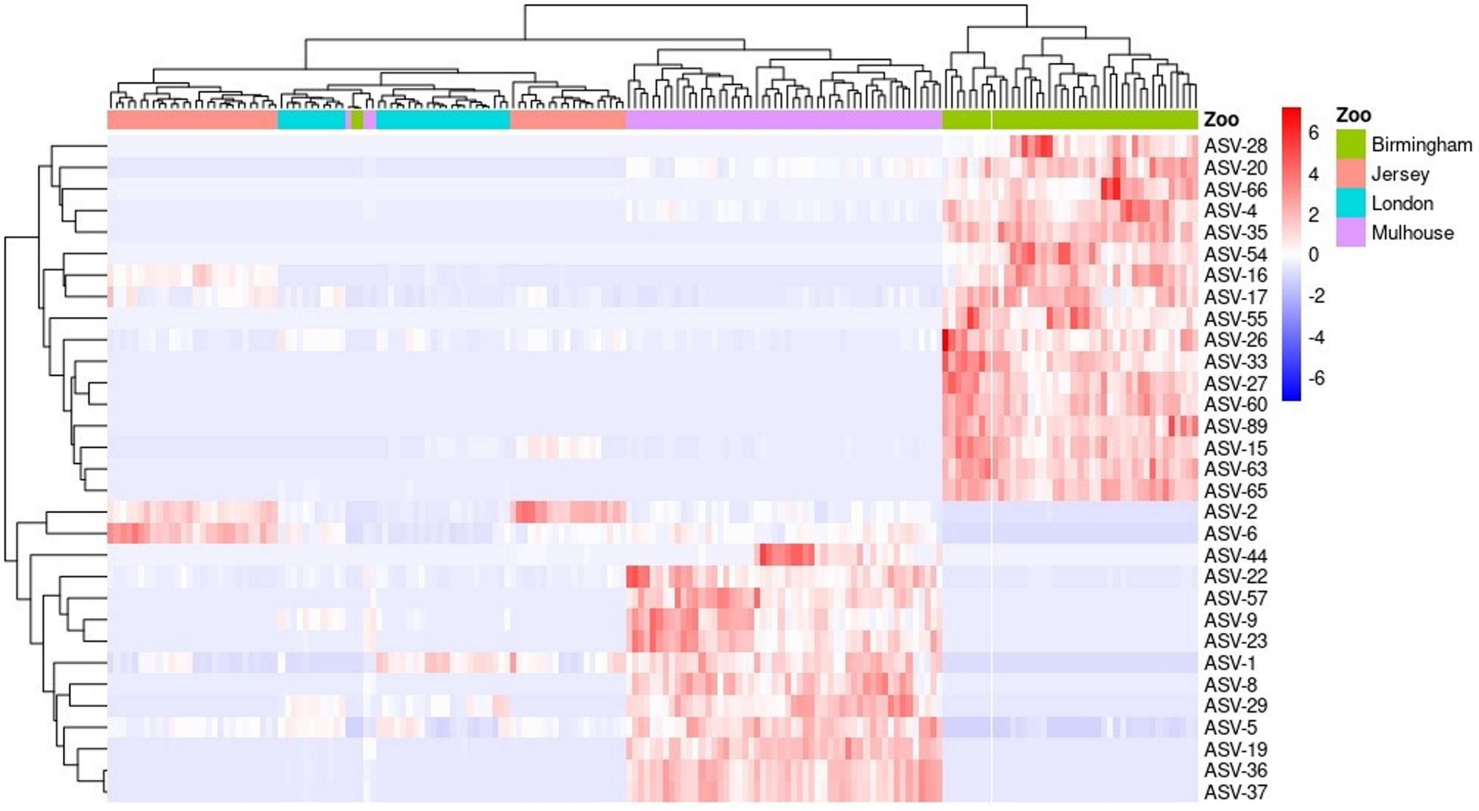
Heatmap and cluster analysis of ASVs identified across the different zoos, with scaled and centered abundances. Sequence variants clustered according to their abundance across samples from different zoological institutions. Amplicon sequence variants reporting a different abundance pattern in one or more sample types were clustered according to their mean variance‐stabilized abundance.

Divisive analysis of hierarchical clustering obtained using variance‐stabilized counts produced two distinct clusters, which show a distinct pattern of ASV abundance that differed among zoological institutions. Clusters 1 and 2 were composed of microbial assemblages that were differently represented across the four zoos. From the heatmap (Figure 5), we can observe that in Cluster 1 (starting from the top), we find a large set of microbial ASVs that best describe Birmingham Zoo, while in the second cluster, we find a set of ASVs that better describe Mulhouse Zoo and, in a smaller part, London and Jersey zoos.

**Figure 6:**
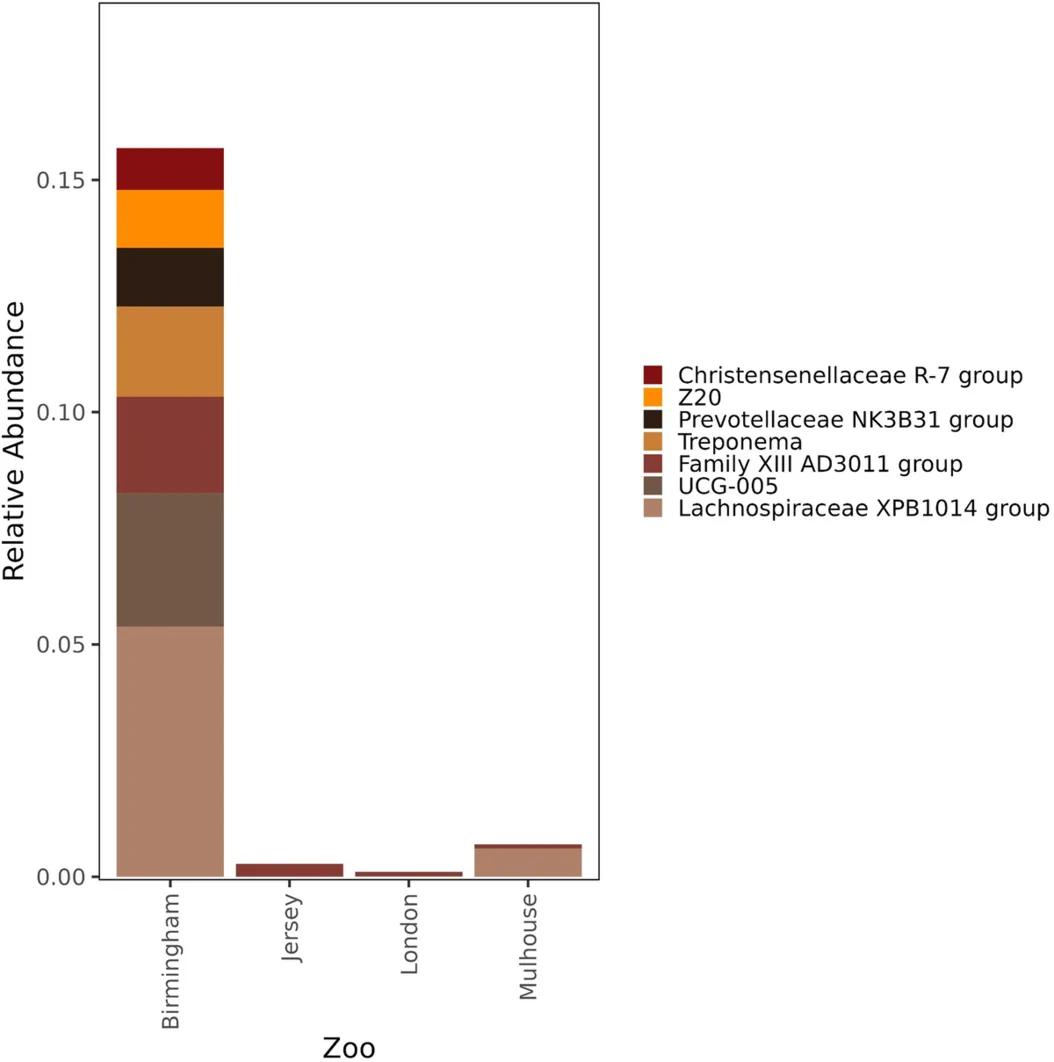
Relative abundance of selected ASVs assigned to bacterial groups best representing the gut microbiota of *Hapalemur alaotrensis* individuals housed at Birmingham Zoo. The microbiota is dominated by members of the *Lachnospiraceae* XPB1014 group, UCG‐005, Family XIII AD3011 group, and *Christensenellaceae* R‐7 group, all involved in fiber fermentation and short‐chain fatty acid production. The substantially higher abundance of these ASVs in Birmingham lemurs suggests a distinct microbial community structure, likely shaped by unique environmental or dietary conditions within this facility.

Considering Cluster 1, we can observe from Figure 6 that among the seven ASVs assigned to bacterial groups that best describe the pair hosted at Birmingham Zoo, we find *Lachnospiraceae* XPB1014, UCG‐005, *Treponema*, and *Prevotellaceae* NK3B31; the presence of these ASVs may indicate a role in fiber digestion, contributing to maintaining a balanced intestinal environment through the production of SCFAs.

**Figure 7:**
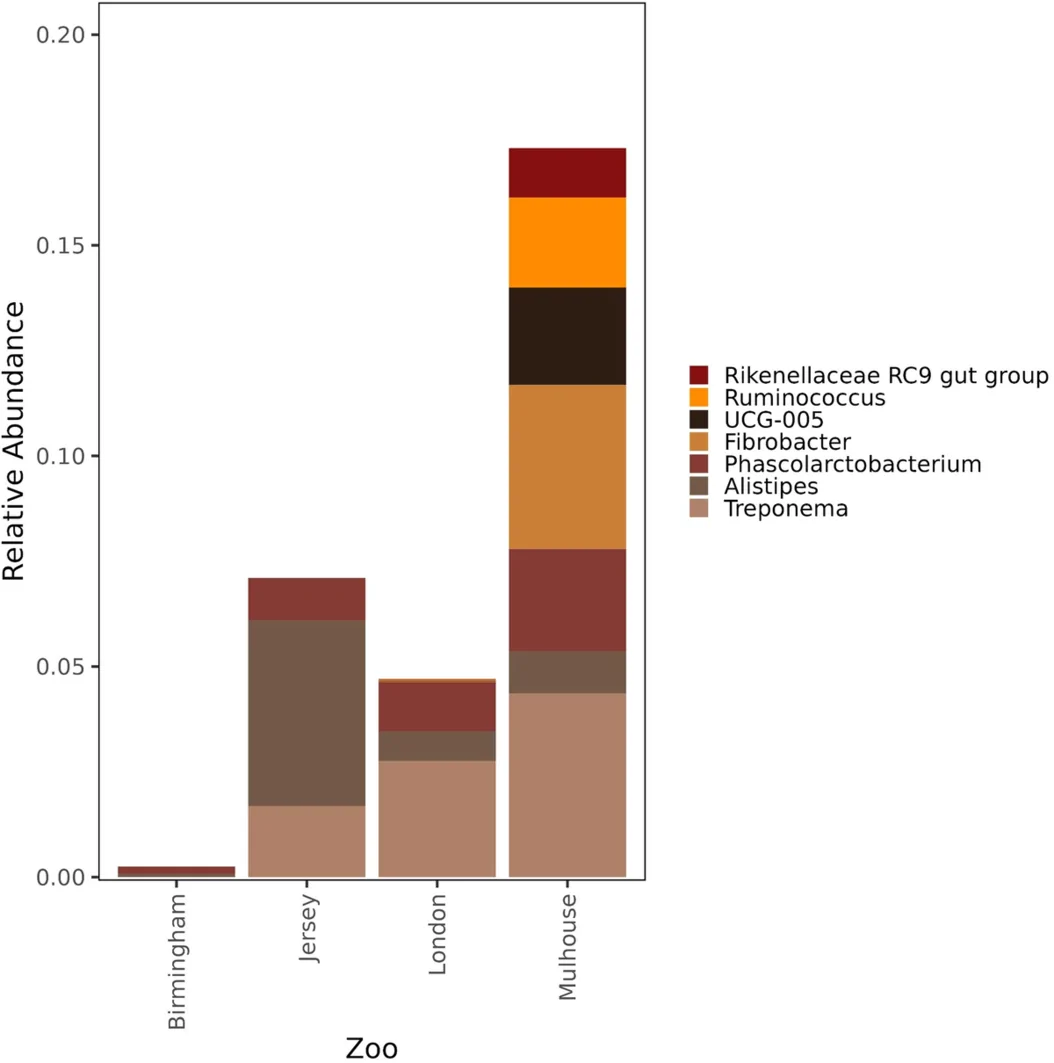
Relative abundance of selected ASVs assigned to bacterial groups best representing the gut microbiota of *Hapalemur alaotrensis* individuals hosted at Jersey, London, and Mulhouse zoos. Samples from Mulhouse exhibited the highest abundance and diversity of fiber‐degrading ASVs, including *Ruminococcus*, *Fibrobacter*, *Phascolarctobacterium*, and *Rikenellaceae* RC9 gut group, all associated with complex carbohydrate fermentation and SCFA synthesis. Jersey and London also featured ASVs assigned to *Alistipes*, *Treponema*, and *Phascolarctobacterium*, taxa linked to fiber digestion and gut health, albeit at different relative abundances.

Cluster 2 obtained from the differential abundance analysis (DESeq2, *p* < 0.05) highlighted distinct compositional profiles for each zoo (Figure 7). In particular, the gut microbiota of lemurs hosted at Mulhouse Zoo was characterized by high relative abundances of ASVs assigned to bacterial groups such as *Rikenellaceae RC9* gut group, *Ruminococcus*, *Fibrobacter*, UCG‐005, and *Phascolarctobacterium*, typically associated with fiber fermentation and SCFA production, along with *Alistipes* and *Treponema*, genera involved in complex carbohydrate breakdown. Samples from London and Jersey also featured ASVs assigned to bacterial genera such as *Alistipes, Treponema*, and *Phascolarctobacterium*, although with different relative abundances.

These patterns contrast with those observed in Cluster 1, where lemurs hosted at Birmingham Zoo displayed a distinct microbiota dominated by ASVs assigned to bacterial groups such as *Christensenellaceae* R‐7 group, *Prevotellaceae* NK3B31, and *Lachnospiraceae* XPB1014 group, also recognized for their role in fiber degradation but suggestive of different dietary substrates.

No significant associations were found between microbiota and the analyzed hormones (cortisol and testosterone) (*p* > 0.05 for all comparisons).

### 3.2 Hormonal Results

As previously reported (Fontani et al. 2025), we did not find any significant effect of enrichment phase on cortisol concentration in the reduced model (*F* = 0.53, df = 2, 170.39, *p* = 0.59). Similarly, our analysis of testosterone concentration revealed no significant effect of the enrichment phase (*χ* ^2^ = 27.8, df = 2, *p* = 0.61).

**Figure 8:**
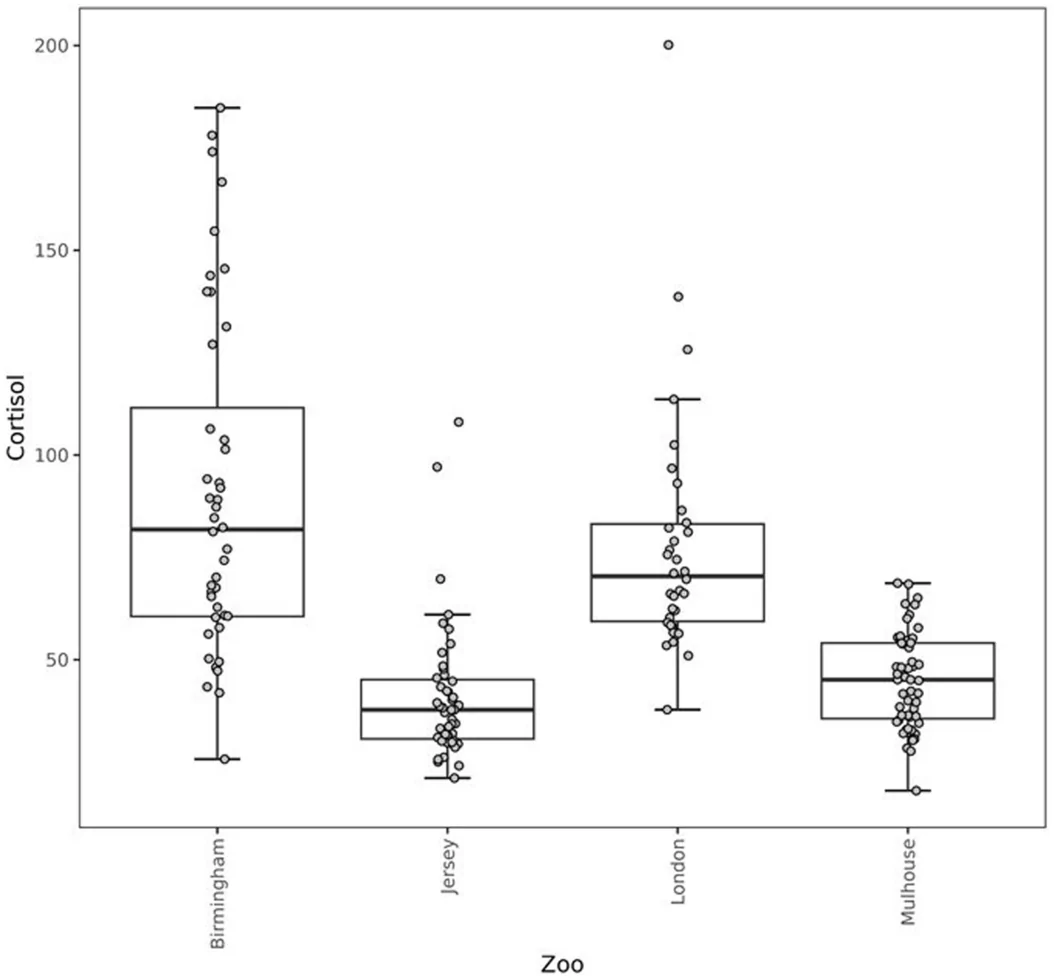
Boxplot of cortisol values across the four zoos.

We then investigated the effects of previously unanalyzed variables (environment and sex) on cortisol and testosterone levels. For cortisol (Figure 8), a significant effect of the environment (i.e., the zoo) was found, with a *p* value of 0.037, which was confirmed by the reduced model (*p* = 2.001*e^−5^). Sex, on the other hand, was found to be nonsignificant in both the full and reduced models (*p* > 0.05).

**Figure 9:**
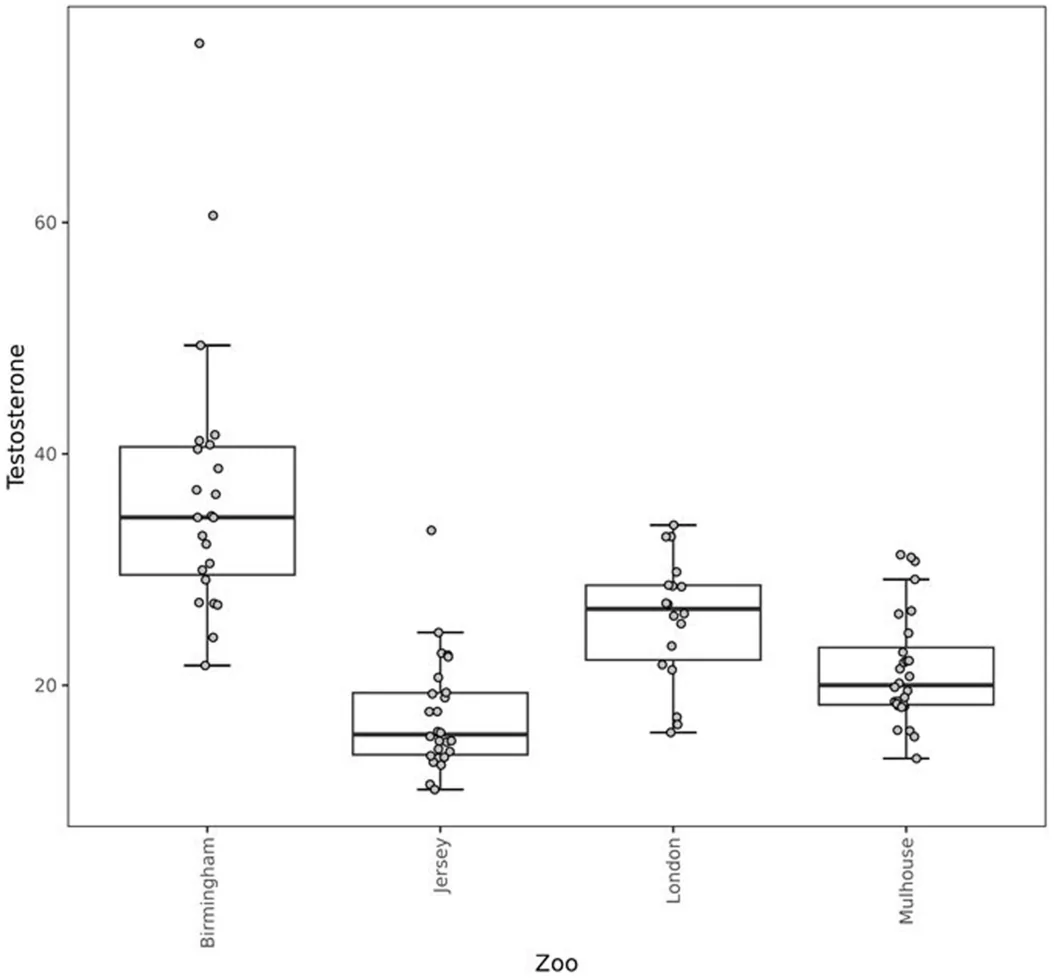
Boxplot of testosterone values across zoos.

Since only one male was present in each zoo, testosterone analysis (Figure 9) was run both using the individual ID as random effect and without the random effect, while using the environment as fixed factor. When running the model including the random effect, the environment was not significantly associated with the hormonal levels. However, when run without the random effect, zoo resulted to be highly significant (*p* = 2.835*e^−15^). This result should be interpreted cautiously, as the effects of zoo and individual identity could not be fully separated.

## 4 Discussion

In this study, we investigated the relationships between gut microbiota composition, hormonal profiles, and host‐specific traits in captive Alaotran gentle lemurs. Building upon our previous work assessing the impact of a new olfactory enrichment on welfare and reproductive potential (Fontani et al. 2025), we aimed to explore how host‐specific characteristics influence microbiome diversity and whether a shared environment facilitates microbial transmission. Furthermore, we sought to explore the potential of simultaneous microbiota and endocrine analyses as a noninvasive approach that may inform future welfare monitoring.

Age was not included among the factors investigated because of the limited number of individuals and the uneven age distribution across study subjects. Among the factors examined, zoo consistently emerged as the strongest factor associated with gut microbiota variation. In alpha diversity analyses, zoo significantly affected both Shannon and Simpson diversity indices, whereas sex was associated only with Shannon diversity. In the unconditioned beta diversity analyses, zoo, sex, and scent enrichment were significantly associated with gut microbiota composition, although the effect of zoo was substantially stronger than that of the other factors. Overall, these findings suggest that both host‐ and environment‐related factors contribute to shaping gastrointestinal microbial communities in captive Alaotran gentle lemurs, with environmental differences among facilities appearing to represent the predominant source of variation.

The association between sex and microbiota diversity and community structure observed in the present study is consistent with previous findings indicating that host sex can contribute to shaping gut microbial communities in both humans and nonhuman primates (Kim et al. 2020; Y. Li et al. 2021). Such sex‐related differences in the microbiota are often attributed to physiological differences associated with endocrine regulation, as well as immune function and metabolism (Vemuri et al. 2019; Yoon and Kim 2021). Similarly, the relatively weak effect of scent enrichment may reflect the short duration of the treatment or the inherent resilience of gut microbial communities over such timescales (Fassarella et al. 2021).

Although the unconditioned model identified significant associations with sex and scent enrichment, these effects were not retained in the conditioned model. This suggests that the observed associations should be interpreted cautiously and may reflect the limited sample size and study design, including the presence of only one male and one female in each facility.

Despite the lack of significant effects in the conditioned model, inter‐zoo differences represented the most consistent pattern across the overall analytical framework, including alpha diversity indices, unconditioned beta diversity analyses, clustering patterns, and ASV‐level taxonomic profiles. Moreover, the clear clustering observed in beta diversity analyses (Figure 4) and the identification of distinct ASV abundance patterns across zoos (Figure 5) indicate that each facility was characterized by a distinct microbial signature. However, although facility‐level differences are a consistent descriptive pattern across several analyses, the formal inference about zoo‐level effects is limited once individual identity is accounted for.

Consistent with the differences observed among zoological institutions, analyses of taxonomic composition revealed variation in the relative abundance of several microbial taxa across study sites. At the phylum level, gut microbial communities were dominated by Firmicutes and Bacteroidota across all institutions, whereas differences among zoos became more evident at lower taxonomic levels. ASVs representing genera such as *Ruminococcus*, *Fibrobacter*, and *Phascolarctobacterium* (some species of which are known for their roles in fermenting plant fibers and producing SCFAs) (Deleu et al. 2021; Lan et al. 2025; Sasaki et al. 2022; Watanabe et al. 2011; Wu et al. 2017) were particularly abundant in lemurs hosted at Mulhouse Zoo. Owing to their capacity to produce SCFAs by fermenting fiber, these taxa may be beneficial for host metabolism and gut health, and may reflect the relatively high availability of fibrous plant material in the diet provided at this institution (Sivaprakasam et al. 2016; Vinelli et al. 2022). Similarly, ASVs representing fiber‐degrading genera such as *Alistipes*, *Treponema*, and *Phascolarctobacterium* were also present in subjects hosted at London and Jersey zoos, although in different relative abundances.

These patterns contrast with the microbial configuration previously identified for Birmingham Zoo, where a dominance of ASVs representing *Christensenellaceae* R‐7 group, *Prevotellaceae* NK3B31, and *Lachnospiraceae* XPB1014 group was detected. Although these taxa are likewise involved in polysaccharide fermentation, their prominence may indicate reliance on dietary substrates differing from those provided in other institutions, potentially including items such as fruits or soft plant material, which are scarce in the natural diet of this species (Mutschler 2002). Such differences in microbial composition are consistent with possible influences of diet and husbandry among host institutions. In particular, the diet provided at Birmingham Zoo includes a higher proportion of commercially prepared feeds and cultivated vegetables, alongside browse, whereas the other institutions place greater emphasis on natural browse and structurally complex plant material. These distinct but nutritionally managed feeding strategies may influence the availability of fermentable substrates in the gut, thereby influencing the relative abundance of ASVs representing specific bacterial taxa involved in carbohydrate metabolism.

Notably, while all participating zoos adhered to modern husbandry standards, the microbial profiles observed suggest that variation in dietary composition among institutions may be reflected in differences in gut microbiota structure. In the wild, Alaotran gentle lemurs feed almost exclusively on Cyperaceae and Poaceae species, avoiding fruits nearly entirely (Mutschler 2002). The presence and abundance of specialist fiber‐fermenting bacteria may thus serve as potential indirect indicators of dietary composition in captivity, highlighting how different, carefully managed dietary approaches can result in distinct but functionally meaningful gut microbiota configurations.

Altogether, our findings highlight the importance of considering both host‐specific and environmental factors when investigating gut microbiota variation in captive primates. However, given the limited number of individuals included in the study, the relative contribution of specific environmental factors, such as diet composition, should be interpreted with caution. Nevertheless, these insights underscore the potential value of microbiome monitoring as a tool to complement husbandry practices and support the conservation and welfare of critically endangered species like the Alaotran gentle lemur.

Regarding hormone analyses, as previously reported (Fontani et al. 2025), we did not detect significant fluctuations in cortisol or testosterone concentrations across the experimental phases. However, significant inter‐zoo differences in cortisol concentrations emerged when sampling locations were compared. In contrast, no sex‐related differences emerged. These results should nevertheless be interpreted cautiously given the limited number of individuals included in the study.

Such differences among study sites are commonly reported in primate research and may reflect a combination of environmental and physiological factors. For example, Bethge et al. (2022) reported seasonal variation in cortisol concentrations in the Malagasy lemur *Lepilemur edwardsi*, with males showing elevated cortisol levels during the mating season, suggesting that both reproductive activity and seasonal environmental conditions can influence HPA‐axis activity. Age has also been reported to influence HPA‐axis activity and responsiveness in primates. In rhesus macaques, significant age‐related changes have been observed in both basal cortisol concentrations and cortisol responses to stress, highlighting the importance of developmental variation in neuroendocrine function (Koch et al. 2014).

When considering sex‐related patterns, differences in cortisol secretion between males and females have been reported in several primate species and often appear to be associated with reproductive activity, social dynamics, and other context‐dependent factors. As noted above, male *L. edwardsi* exhibited elevated cortisol concentrations during the mating season, whereas females showed no seasonal changes, suggesting a sex‐specific response linked to reproductive activity (Bethge et al. 2022). A different pattern was reported by Garber et al. (2020), who found higher cortisol concentrations in wild female common marmosets (*Callithrix jacchus*) than in males, a pattern associated with the combined effects of reproductive demands and social competition among females. By contrast, Phillips et al. (2018) found no evidence of adult sex‐based differences in cortisol concentrations in captive common marmosets housed in stable, nonreproducing groups consisting of a single adult male and a single adult female. The authors suggested that the absence of reproductive competition and other social and physiological stressors may have contributed to the lack of detectable sex differences. Similarly, the Alaotran gentle lemurs included in the present study were housed as stable male–female pairs under managed zoo conditions and were not reproductively active during the study period. As such, the absence of detectable sex‐related differences in cortisol concentrations may reflect the limited social and reproductive pressures experienced by the study animals.

By contrast, testosterone concentrations were not significantly associated with zoo once individual variation was taken into account. However, interpretation of this result is limited by the fact that only one male was present in each institution, preventing a clear separation of zoo effects from individual‐specific variation. Testosterone secretion in primates is known to be influenced by multiple biological and social factors, including age, reproductive status, seasonality, and social context (Beehner et al. 2009; Emery Thompson et al. 2012; Wobber et al. 2010). Consequently, variation among individual males may reflect a combination of physiological and social factors that cannot be disentangled within the framework of the present study.

Finally, no correlations were detected between hormone levels and microbiota profiles. The relationship between endocrine activity and gut microbiota composition is currently understood as bidirectional within the framework of the microbiota–gut–brain axis, where hormonal fluctuations may influence microbial communities, while the gut microbiota itself can modulate neuroendocrine signaling (Ohara and Hsiao 2025; So and Savidge 2022; Stopińska et al. 2021). In the present study, olfactory enrichment was hypothesized as a potential modulator of physiological state that could be reflected in both hormone levels and gut microbiota composition. However, given the absence of significant hormone fluctuations associated with the experimental phases, together with the limited sample size and sampling duration, potential associations between endocrine activity and microbiota variation may not have been detectable under the experimental conditions of the present study.

## 5 Conclusions

This exploratory study reports facility‐associated microbiome variation in a small sample of zoo‐housed Alaotran gentle lemurs, with no detected hormone–microbiome association under the present sampling conditions. Nevertheless, among the factors examined, differences among zoological institutions represented the most consistent pattern across the analytical approaches, suggesting that environmental and husbandry‐related factors, including diet, may be associated with gut microbial communities. Although we did not find a correlation between endocrine patterns and microbial profiles, given the increasing evidence supporting the gut–brain axis in primates, this area warrants further investigation through studies employing larger sample sizes and longitudinal designs. Despite the limited effects of scent enrichment on the gut microbiota and the absence of enrichment‐related hormonal changes, our results suggest that integrating microbiota characterization with endocrine assessments may provide a more comprehensive framework for evaluating physiological status in captivity and may help inform future welfare monitoring strategies. Such combined, noninvasive approaches may ultimately support more informed management decisions and contribute to the long‐term conservation of this critically endangered species.

## 6 Research Limitations

We acknowledge several limitations that may have influenced the outcomes of this study. First, the relatively small sample size, constrained by the limited number of captive individuals of *H. alaotrensis*, inevitably reduced the statistical power and the generalizability of the results. Moreover, while the scent enrichment implemented was carefully designed and, as reported in Fontani et al. (2025), produced certain behavioral effects, it may not have been sufficiently impactful to induce measurable changes in hormonal or microbiota levels. Additionally, potential environmental sources of microbial variation related to animal husbandry cannot be excluded. Cross‐contamination through keepers' footwear, shared food preparation areas, or other aspects of routine management may have contributed to bacterial differences observed in fecal samples. Finally, the selection of study individuals based on low breeding potential may have introduced a further source of bias, particularly with respect to testosterone levels and, more broadly, aspects of physiological health, which should be considered when interpreting the results.

## 7 Future Directions

Given the zoo‐specific variation in gut microbiota composition observed in this study, our next step will be to collect fecal samples from wild Alaotran gentle lemur populations in Madagascar to enable direct taxonomic and functional comparisons of microbiome composition between captive and wild individuals. We also plan to collect soil samples from wild habitats to characterize environmental microbial reservoirs and potential sources of host‐associated taxa, thereby clarifying environmental contributions to gut community assembly. In addition, we intend to isolate yeasts from fecal samples to establish a reference collection that will support future genomic and functional studies, with the broader aim of elucidating host–bacterial microbiota interactions, while also exploring the potential role of yeasts as an additional component of the gut ecosystem, and the functional implications for host physiology and welfare. These investigations may contribute to evidence‐based improvements in the health monitoring and management of zoo‐housed *H. alaotrensis* and support long‐term population sustainability.

## Author Contributions

**Sara Fontani:** study conception and design, training of research assistants in data collection and endocrinological laboratory work, funding acquisition, project management, writing original draft. **Agnese Gori:** microbiology laboratory work, writing original draft. **Benedetta Cerasuolo:** microbiology laboratory work. **Sonia Renzi:** microbiology laboratory work. **Aldo D'Alessandro:** statistical analysis, writing original draft. **Gale Glendewar:** study conception and design, assistance in data collection. **Rachel Cowen:** study conception and design, assistance in data collection. **Duccio Cavalieri:** study conception and design, reviewing original draft, funding acquisition, supervision. **Stefano Vaglio:** study conception and design, reviewing original draft, funding acquisition, project administration, supervision. All authors read and approved the final manuscript.

## Ethics Statement

This study adhered to both institutional and international guidelines for the care and use of captive animals, employing noninvasive methods for data collection. It was performed in accordance with the Animals (Scientific Procedures) Act 1986 Amendment Regulations (SI 2012/3039) and conformed to Directive 2010/63/EU. It complied with the ARRIVE guidelines for the ethical treatment of nonhuman primates and was conducted in accordance with CITES regulations. It received approval from the Life Sciences Ethics Committee at the University of Wolverhampton (UK) (REC Number LSEC/202021/SV/52), as well as from the ethics committees at Jersey Zoo (Channel Islands), Parc Zoologique & Botanique de Mulhouse (France), Birmingham Wildlife Conservation Park, and the Zoological Society of London (ZSL) London Zoo (UK), and complied with the American Society of Primatologists Principles for the Ethical Treatment of Nonhuman Primates.

## Conflicts of Interest

The authors declare no conflicts of interest.

## Supporting information


Supporting File

## Data Availability

The data sets are available on the European Nucleotide Archive (ENA) repository under Accession Code PRJEB97323.
