# Supplementary material for: Microbial Variability in Zoo‐Housed Alaotran Gentle Lemurs: Host‐Associated Factors and Environmental Influences
Source: Am J Primatol. 2026 Aug 26;88(8):e70202. doi: 10.1002/ajp.70202 (PMC13512788; doi:10.1002/ajp.70202)
Supplement: Supplementary file 1 [file AJP-88-e70202-s001.pdf]

Supplementary Table S1. Dietary composition of zoo-housed Alaotran gentle lemurs (*Hapalemur alaotrensis*) across participating zoological institutions

Birmingham Zoo

AM Feed
16g Graze-On
10g Exotic Leaf Eater Pellets
20g Carrot (chopped)
20g Cucumber (chopped)
20g Chicory (chopped)

PM Feed
10g Graze-On
40g Exotic Leaf Eater Pellets
20g Carrot (chopped)
20g Chicory (chopped)
20g Cucumber (chopped)

+ browse ad lib when possible (bamboo, bramble, willow, sedge grass)

Mulhouse Zoo

Feeding program

08.00hr Mazuri leaf-eater primate pellets soaked in 10% Vitapualia M (10mL Vitapaulia M for 1L of water) 20g

11.00hr Bamboo and half of the meal from 18.00hr

16.00hr Bamboo

18.00hr Apples 40g

Other fruits + vegetables 80g

Simial powder 5g

Lettuce/endive/cabbage

Diet composition in grams AF per animal per day

Constant: Apple, cooked carrot, cucumber, courgette, aubergine, pepper, chicory, lettuce.

Frequent: Banana, grapes, kiwi

Less frequent: Nectarine, peach, cherry, fig, dried fig, strawberry, pineapple, grapefruit, carambola, red cabbage, radish, broccoli.

In April-May, when the bamboo starts to grow, young shoots of bamboo are also given

to the Alaotran gentle lemurs, in combination with bamboo leaves.

Jersey Zoo

Diet per animal

BREAKFAST FEED 8.30-9am

15g leaf-eater pellet, lightly soaked with water + sprinkle Vionate powder + 2 thin slices of carrot (no carrot for overweight animals and pellet reduced to 11g)

FORAGE FEED Midday

One of the below:

- Bamboo – majority shoots/very young leaves
- Willow - **Not weeping willow, corkscrew or goat willow**.
- Grass
- Miscanthus (Elephant Grass)
- Phragmites (reeds)
- Chicory

PM FEED

- Couple of leaves of chicory
- ¼ tomato
- 3 small pieces celery
- 3 small quarters of cucumber
- 2 small pieces of carrot
- (1ml 'Collo-Cal D' in soaked leaf-eater pellet for nursing mothers -up to 6 months)

London Zoo

**DIET PRESENTED (average per animal per day)**	
**Food item**	**Weight fed (g)**
DK Zoological leaf eater pellet	5 (was up to 10g)
Readi grass (Friendship Estates)/Just grass	100
Leafy green vegetables (Chicory or little gem)	100 (winter 200g)
Other vegetables (cucumber or celery)	20
Grasses (phragmites, miscanthus, arundo, fox tail millet, & papayrus)	Fed daily in summer & as available in winter.

**Additional dietary notes**
During summer, fresh browse and grasses account for approximately 80% of the diet offered. Intake of these varies widely; the animals are selective, preferring new growth and are reluctant to consume older matter. During winter, browse availability decreases and animals receive a greater proportion of vegetables and Readi grass. Pellet intake is variable on a temporal and individual basis.<br><br>Pregnancy & lactation – add Nutrobal to veg.
